# Shared genetic architecture between mental health and the brain functional connectome in the UK Biobank

**DOI:** 10.1186/s12888-023-04905-7

**Published:** 2023-06-23

**Authors:** Daniel Roelfs, Oleksandr Frei, Dennis van der Meer, Elleke Tissink, Alexey Shadrin, Dag Alnaes, Ole A. Andreassen, Lars T. Westlye, Tobias Kaufmann

**Affiliations:** 1 NORMENT Centre, Division of Mental Health and Addiction, Oslo University Hospital & Institute of Clinical Medicine, University of Oslo, Oslo, Norway; 2 School of Mental Health and Neuroscience, Faculty of Health, Medicine, and Life Sciences, Maastricht University, Maastricht, The Netherlands; 3 Department of Complex Trait Genetics, Center for Neurogenomics and Cognitive Research, Vrije Universiteit Amsterdam, Amsterdam Neuroscience, Amsterdam, 1081 HV The Netherlands; 4 Bjørknes College, Oslo, Norway; 5 Department of Psychology, University of Oslo, Oslo, Norway; 6 Department of Psychiatry and Psychotherapy, Tübingen Center for Mental Health, University of Tübingen, Tübingen, Germany; 7 German Center for Mental Health (DZPG), Tübingen, Germany

**Keywords:** Computational psychiatry, Mental health, Brain connectivity, Genetics, Multivariate GWAS

## Abstract

Psychiatric disorders are complex clinical conditions with large heterogeneity and overlap in symptoms, genetic liability and brain imaging abnormalities. Building on a dimensional conceptualization of mental health, previous studies have reported genetic overlap between psychiatric disorders and population-level mental health, and between psychiatric disorders and brain functional connectivity. Here, in 30,701 participants aged 45–82 from the UK Biobank we map the genetic associations between self-reported mental health and resting-state fMRI-based measures of brain network function. Multivariate Omnibus Statistical Test revealed 10 genetic loci associated with population-level mental symptoms. Next, conjunctional FDR identified 23 shared genetic variants between these symptom profiles and fMRI-based brain network measures. Functional annotation implicated genes involved in brain structure and function, in particular related to synaptic processes such as axonal growth (e.g. NGFR and RHOA). These findings provide further genetic evidence of an association between brain function and mental health traits in the population.

**Supplementary Information:**

The online version contains supplementary material available at 10.1186/s12888-023-04905-7.

## Introduction

Psychiatric disorders are complex, with a polygenic architecture, and large degree of overlapping symptoms and risk factors. Both imaging and genetics studies have shown numerous but small associations between brain phenotypes, psychiatric disorders, and genetics, such as schizophrenia (SCZ) [1–3], bipolar disorder (BIP) [4, 5], major depressive disorder (MDD) [6, 7], and anxiety disorder (ANX) [8–10]. Interactions between various brain phenotypes and genetics have been reported across structural [11, 12] and functional [2, 7, 10] imaging modalities.

We have recently deployed a multivariate analysis to study the genetic architecture of brain functional connectivity, revealing genetic variants associated with functional brain connectivity as well as variance in brain activity over time [13]. The results showed meaningful overlap with psychiatric disorders, pointing at synapse-related pathways among the biological processes shared between disorders and brain function [13].

Previous studies have shown widespread phenotypic and genetic overlap between psychiatric disorders [14–18]. In addition, patients within a diagnostic category can display a wide variety of symptoms. This heterogeneity complicates both diagnosis and therapeutic response due to overlapping symptoms and generally low specificity of diagnostic features [19, 20]. While the mental health of any individual in the population varies over the course of a lifetime, most will not meet diagnostic criteria for a psychiatric disorder [21, 22]. In order to capture the variance encompassing psychiatric symptoms that is lacking in traditional case-control studies, one can use population-level mental health questionnaires as implemented in the UK Biobank [23]. This facilitates analyses using the continuous scales which enable data-driven clustering methods to extract different profiles each capturing a separate domain relevant to mental health in a sample without individuals diagnosed with a psychiatric disorder, taking advantage of larger sample sizes. Using independent component analysis (ICA), we have previously derived 13 mental health profiles from UK Biobank data, and showed that, although phenotypically independent (by design) they nonetheless share genetic underpinnings [24].

Here, we aimed to uncover the genetic architecture of mental symptoms and identify shared genetic loci with neurobiological processes related to brain function. Using multivariate analysis [25], we generated multivariate genome-wide association statistics across our previously identified 13 population-level mental health profiles [24]. This allowed us to identify new gene variants associated with mental health symptoms and traits such as psychosis, depression, and anxiety in the UK Biobank sample not captured in a univariate analysis. Further, we combined this multivariate genetic profile of mental health with GWAS summary statistics of 7 psychiatric disorders and with our previously identified multivariate profiles of functional brain connectivity and variance in brain activity over time [24]. This research aims to provide insight into the biological underpinnings of mental health symptoms.

## Methods

### Sample and exclusion criteria

We utilized data from the UK Biobank [26] with permission no. 27,412. All participants provided signed informed consent before inclusion in the study. The UK Biobank was approved by the National Health Service National Research Ethics Service (ref. 11/NW/0382). We previously used data from the online follow-up questionnaire on mental health to define 13 phenotypically independent profiles relevant for mental health [24]. We utilized summary statistics from a previous study [13] in which we deployed the UK Biobank imaging resource with 30,701 participants of White British ancestry aged 45–82 years (52.8% females). In this study, we performed multivariate genome wide association analyses of functional connectivity measures (partial correlations as a measure of edge strength between brain networks, and temporal node variance as a within network measure) using the Multivariate Omnibus Statistical Test (MOSTest). The resulting summary statistics were used as a starting point in the current study. In another previous study, we performed an independent component analysis of 136 items from a mental health questionnaire of 117,611 UK Biobank participants aged 47 to 80 (56.2% female) [24]. The derived 13 components reflect mental health profiles for different mental health domains and were here fed into MOSTest, to derive a multivariate GWAS of mental health. For this study we deployed the summary statistics from these previous studies.

### Image acquisition and pre-processing

The processing pipeline for imaging data used for the multivariate GWAS is described in Roelfs et al. [13]. In short, images were acquired using 3T Siemens Magnetom Skyra scanners with a 32 channel head coil (Siemens Healthcare GmbH, Erlangen, Germany) at four different sites in the UK. The fMRI data was recorded using a gradient-echo echo planar imaging sequence with x8 multislice acceleration (TR: 0.735s, TE: 39ms, FOV: 88 × 88 × 64 matrix, FA: 52°) with a voxel size of 2.4 × 2.4 × 2.4 mm. Data is processed by the UK Biobank team following the protocol described in Alfaro-Almagro et al. [27].

### Multivariate genome-wide analysis

In this study we applied MOSTest to the phenotypic data from the ICA decomposition described in Roelfs et al. [24]. MOSTest deploys the univariate test-statistics for each SNP and computes a multivariate test statistic through single random permutations of the genotype vector. Standard preprocessing of the genotyping array data is described in Bycroft et al. [28] We further preprocessed the data by filtering out individuals with more than 10% missingness rate, SNPs that were missing in more than 5% of genomes, and dropping of SNPs failing the Hardy-Weinberg threshold of 1 × 10e-9 and removing variants failing the MAF threshold at 0.005. We performed positional gene mapping using Functional Mapping and Annotation (FUMA) [29]. We also used a built-in tool to follow up these gene mapping analyses using MAGMA to connect the identified genes with tissue types [30]. For FUMA we used the default settings with a reference panel from the 1000G Phase3 genome project with subjects with a White European ancestry. The MAF threshold was 0.005, loci were defined as at least 250 kb long. A gene was mapped when it was at most 10 kb removed from the SNP. We analyzed gene sets using the reactome toolbox [31] to identify biological processes associated with the genes associated with the summary statistics identified by FUMA.

### Pleiotropy-informed conjunctional false discovery rate

In order to quantify the degree of genetic overlap and identify shared genetic loci between the mental health profiles and the imaging features we deployed the pleiotropy-informed conjunctional false discovery rate (conjFDR) through the pleioFDR toolbox [32]. We used the default settings for conjunctional FDR. SNPs were pruned if they had an LD score higher than 0.2. Cross-trait enrichment was avoided by ensuring the input summary statistics had no sample overlap. For this, we removed any individuals included in the fMRI analyses from the MOSTest analysis of mental health. One of the advantages of conjFDR is that it can identify shared genetic loci regardless of effect direction and effect size, a feature that is useful when working with multivariate measures where effect direction might be lacking.

## Results

**Fig. 1:**
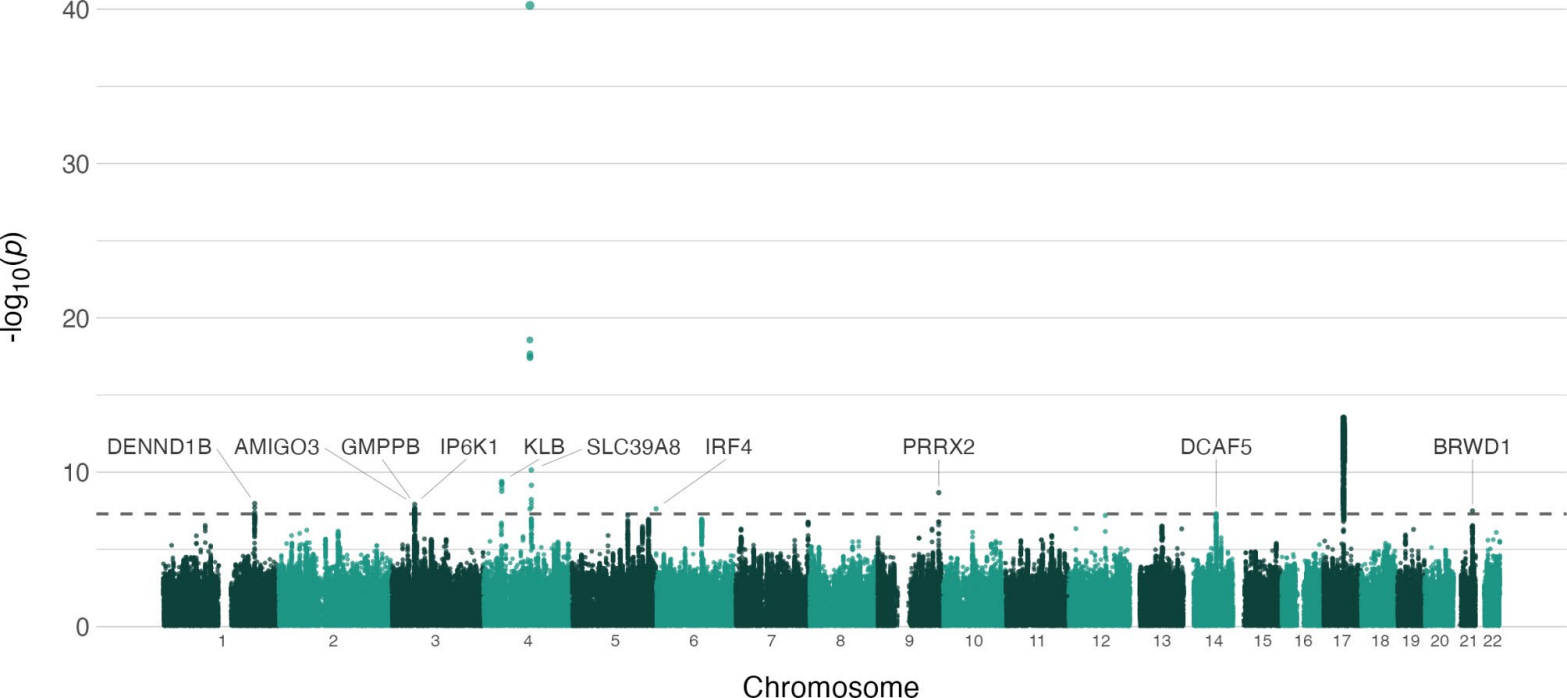
Manhattan plot of multivariate genome-wide association statistics for mental health. Manhattan plot showing the multivariate genome-wide association of our multivariate measure of mental health. We identified 10 loci associated with the multivariate genome-wide association statistics for mental health.

MOSTest revealed 10 significant (P < 5e-8) loci across the 13 previously identified profiles of mental health (Fig. 1). FUMA and its positional mapping tool revealed 48 genes associated with these loci (See Suppl. Table 1), that were linked by MAGMA to (among other anatomical structures and tissues) a number of brain structures such as the cerebellum and amygdala (Suppl. Figure 1). Among the identified genes, those mapped from the strongest GWAS loci were *ADH1B* and *ADH5* (chromosome 4) and *CRHR1* (chromosome 17).

In order to identify shared genomic loci between the mental health profiles and psychiatric disorders, we used GWAS summary statistics from prior case-control studies including schizophrenia (SCZ) [1], bipolar disorders (BIP) [4], major depression (MD) [6], attention-deficit hyperactivity disorder (ADHD) [33], autism spectrum disorder (ASD) [34], post-traumatic stress disorder (PTSD) [35], and anxiety (ANX) [8], see also Suppl. Table 2. First, we compared the gene set from the multivariate mental health genome-wide association statistics with the gene set from each of the psychiatric disorders. Here we found 35 overlapping genes, 29 with SCZ, 7 with BIP, and 1 with ADHD (see Suppl. Table 3). It is important to note that the 7 overlapping genes with BIP were mapped from only 2 separate loci. Next, we extracted the loci from each case-control GWAS (202 in total) and assessed whether each locus was significant in the multivariate genome-wide association statistics for mental health profiles as well. Of the 202 loci significant in any of the disorders, one showed genome-wide significance at P < 5e-8 and 122 showed nominal significance at P < 0.05 only in the multivariate genome-wide association statistics for mental health profiles. This may potentially indicate shared but small effects, however, caution is warranted given the lack of adjustment for multiple comparison in these findings.

Next, we explored the genetic overlap between the mental health profiles and the psychiatric disorders through the conjunctional false discovery rate (conjFDR) [32, 36] which leverages pleiotropy between two phenotypes to estimate shared genetic determinants. ConjFDR allows for the discovery of shared genetic determinants even when those loci are not genome-wide significant in either of the traits in the analysis. Through conjFDR we identified 35 overlapping loci in total between the multivariate genome-wide association statistics for mental health profiles and psychiatric disorders. We found 10 overlapping loci between the multivariate measure of mental health profiles and BIP, 8 overlapping loci with both MD and ADHD, 5 overlapping loci with SCZ, and 4 overlapping loci with autism (see Suppl. Figure 2). FUMA identified 89 genes associated with these loci (See Suppl. Table 4). We found no overlapping loci between the mental health profiles and ANX or PTSD, which may be related to the limited power in these GWASs (see Suppl. Table 2).

**Fig. 2:**
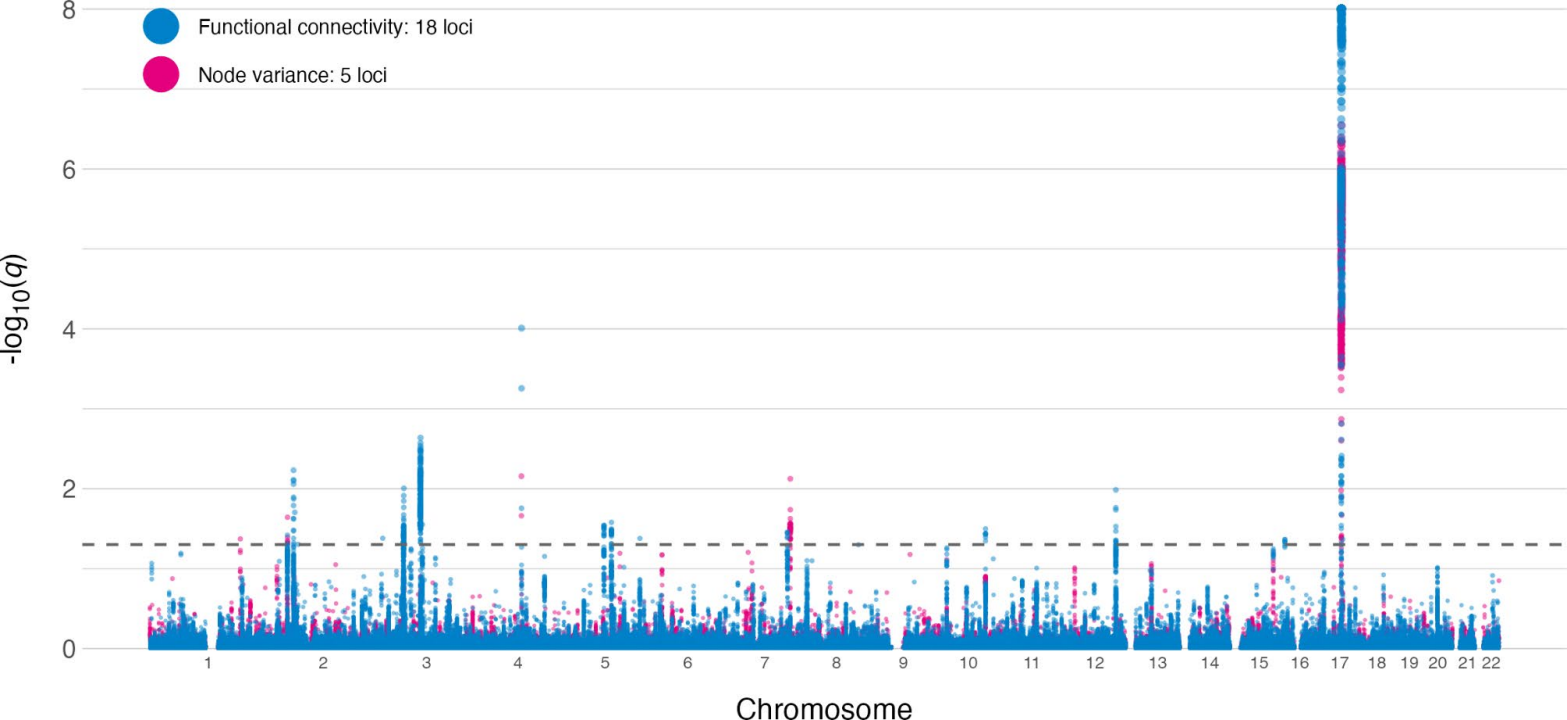
Manhattan plot of conjFDR between multivariate genome-wide association statistics for mental health and the brain functional connectome Association strength per locus is depicted as q-value from the conjunctional FDR. Values for FC and node variance are shown in the same figure with separate colors

We then calculated the number of shared genetic loci between the multivariate genome-wide association statistics for mental health profiles and the two multivariate measures of the brain functional connectome using conjFDR. The GWAS summary statistics from our previous study of brain function [13]. In short, these two summary statistics reflect the multivariate genetic fingerprint of the partial correlation matrix of the connection strength between different areas of the brain and temporal variance inside each brain network [13]. In contrast to the prior study in which we identified genetic overlap between brain function and psychiatric disorders, we here investigated overlaps with the multivariate genome-wide association statistics for mental health to investigate if this approach captures associations not revealed through case-control GWAS approaches. Figure 2 shows two Manhattan plots of the conjunctional FDR analyses between both functional connectivity and node variance with the multivariate GWAS on the multivariate genome-wide association statistics for mental health profiles. Genetic signal was adequate (See Suppl. Figure 3). The multivariate genome-wide association statistics for mental health profiles shared 18 loci with functional connectivity and 5 with node variance. A full list of genes associated with the (in total) 23 unique shared loci between the multivariate summary statistics and FC is presented in Suppl. Table 5. The number of overlapping loci between the brain functional connectome and the multivariate genome-wide association statistics for mental health profiles (18 for FC, 5 for node variance) was generally larger than the number of shared loci between the brain functional connectome and the psychiatric disorders (with the exception of SCZ) identified in our previous study [13]. When we mapped the genes from these loci using FUMA and tested for enrichment in gene-sets using the reactome toolbox [31] we found that the genes associated with these shared loci are involved in a number of neurobiologically relevant processes such as axonal growth regulation (NGFR and RHOA) and regulation of transcription factors related through MECP2 (MEF2C, see Suppl. Table 6).

## Discussion

In this study we identified a number of loci associated with multivariate genome-wide association statistics for mental health profiles and found overlapping loci with the measures of brain function and psychiatric disorders. Using MOSTest we were able to leverage the phenotypic overlap between different mental health profiles to identify new loci associated with a multivariate measure of mental health. Genes associated with these loci showed regional expression in different parts of the brain (e.g. cerebellum, amygdala).

Our analysis using conjFDR revealed a number of shared loci and genes between the multivariate genome-wide association statistics and the psychiatric disorders. This demonstrates the shared genetics between psychiatric symptoms regardless of clinical diagnosis, emphasizes the utility of using population-level phenotypes to investigate variance in mental health profiles, and highlights the advantage in leveraging pleiotropy between complex phenotypes to boost discovery. We found shared genes with all but two case-control GWAS (ANX, PTSD), which also had the two smallest sample sizes, which may reflect insufficient power to detect an effect [37], or may indicate the absence of an effect with those disorders. The largest overlap was with SCZ, which shared 29 genes in the geneset with the multivariate measure. Future sample increases in the case-control GWAS may reveal shared genetics with other complex traits, including population based mental health phenotypes and brain imaging features.

We also identified a number of overlapping loci between mental health profiles and fMRI measures of brain function, including 18 shared loci with functional connectivity and 5 shared loci with node variance. The higher number of shared loci for functional connectivity might be partially explained by the number of phenotypes in each composite measure. While the functional connectivity GWAS comprises 210 measures, i.e. partial correlations between 21 brain nodes, the node variance GWAS encompasses only the temporal variance in each node. It is possible that the number of phenotypes included in the multivariate analysis can affect the discovery [25]. Both the functional connectivity and node variance summary statistics had the same sample size (N = 30.701). For our analyses this means that the difference in their overlap with the multivariate genome-wide association statistics for mental health is due to either the discrepancy in the number of features contained within the composite measure, or alternatively because of different biological processes underlying both measures. The measures differ in that functional connectivity refers to the correlation between brain networks (edge strength), which is possibly governed by different processes than the temporal variance in activity within brain networks. Overall, we found a number of genes associated with the shared loci that are involved in biologically relevant processes such as axonal growth and energy transport (See Suppl. Table 6). Although more thorough functional analysis is necessary, this could suggest that axonal growth processes is a shared feature between brain connectivity and mental disorders, which would be in line with previous evidence linking axonal growth with both processes independently [38–40].

The two conjunctional analyses with fMRI measures and the multivariate genome-wide association statistics for mental health each showed a number of overlapping loci. Not all shared loci were unique, this can be partially explained by the definition of the brain networks in our analyses. The functional connectivity and the node variance measures use the same 21 nodes, and, ultimately, the two measures reflect different properties of the same time series. We found that the number of overlapping loci between the multivariate genome-wide association statistics for mental health and the brain functional connectome was generally larger than findings from our previous study highlighting shared genetic loci between psychiatric disorders and the brain functional connectome. This may partly be due to the larger sample size of the multivariate measure of mental health, but it could also reflect that the multivariate genome-wide association statistics capture genetic variance more generally related to the brain functional connectome. We found little direct overlap between loci of these two GWAS’ separately, which highlights the discovery boost advantage of using conjFDR in phenotypes with generally low heritability.

The main implication from our findings is that we can identify shared genetic variants between a multifactorial measure of mental health in an undiagnosed population sample and fMRI-based measures of brain functional connectivity. Several limitations should be considered. First, the data were obtained from a middle-aged and older White British population, which limits the generalizability of the findings. Further, the mental health questionnaires are self-administered, so the data is vulnerable to various response and self-selection biases [41]. We excluded individuals with a psychiatric diagnosis in our independent component analysis in order to maximalize the population variance and to mitigate the influence of a smaller number of individuals with a (diagnosed) psychiatric condition. This results in a healthier sample, lower variance on a number of severe symptom domains and possible survivor biases [24]. This bias also extends to the imaging data, where participants are reportedly healthier than the general population [42]. Further, MOSTest currently lacks effect direction. This complicates further analyses such as genetic correlations that require reliable effect directions. Nonetheless, FUMA and MAGMA revealed brain structures associated with these mapped genes, such as the cerebellum, amygdala, and various parts of the cortex, which have been linked to psychiatric disorders and symptoms. To what degree these shared genes can explain shared clinical characteristics such as symptoms is an important and relevant issue that needs to be answered in future studies.

In conclusion, our multivariate GWAS on 13 mental health symptom profiles showed a number of shared genetic loci with two fMRI-measures reflecting brain function and connectivity. This research shows that genetic overlap between mental health symptom profiles and brain functional connectivity can be linked to, among other processes, axonal growth regulation (through NGFR and RHOA) and regulation of MECP2 transcription factors (through MEF2C). This provides further genetic evidence of an association between brain function and mental health traits in the population.

## Electronic supplementary material

Below is the link to the electronic supplementary material.

Supplementary Material 1: Supplementary Figures and Tables

## Data Availability

All data used in this study are part of the publicly available UK Biobank initiative (https://www.ukbiobank.ac.uk/). Summary statistics for the disorders are publicly available through their respective consortia. The summary statistics for the multivariate analyses will be shared on GitHub upon acceptance. Code will be made publicly available via GitHub (https://www.github.com/norment/open-science) upon acceptance of the manuscript.

## References

[1] Pardiñas AF (2018). Common schizophrenia alleles are enriched in mutation-intolerant genes and in regions under strong background selection. Nat Genet.

[2] Pettersson-Yeo W, Allen P, Benetti S, McGuire P, Mechelli A (2011). Dysconnectivity in schizophrenia: where are we now?. Neurosci Biobehav Rev.

[3] Trubetskoy V (2022). Mapping genomic loci implicates genes and synaptic biology in schizophrenia. Nature.

[4] Mullins N (2021). Genome-wide association study of more than 40,000 bipolar disorder cases provides new insights into the underlying biology. Nat Genet.

[5] Vai B, Bertocchi C, Benedetti F (2019). Cortico-limbic connectivity as a possible biomarker for bipolar disorder: where are we now?. Expert Rev Neurother.

[6] Wray NR (2018). Genome-wide association analyses identify 44 risk variants and refine the genetic architecture of major depression. Nat Genet.

[7] Zhuo C (2019). The rise and fall of MRI studies in major depressive disorder. Transl Psychiatry.

[8] Otowa T (2016). Meta-analysis of genome-wide association studies of anxiety disorders. Mol Psychiatry.

[9] Stein MB (2009). Neurobiology of generalized anxiety disorder. J Clin Psychiatry.

[10] Thompson PM (2020). ENIGMA and global neuroscience: a decade of large-scale studies of the brain in health and disease across more than 40 countries. Transl Psychiatry.

[11] Cheng W (2021). Genetic Association between Schizophrenia and cortical brain surface area and thickness. JAMA Psychiatry.

[12] Smeland OB (2018). Genetic overlap between Schizophrenia and volumes of Hippocampus, Putamen, and intracranial volume indicates Shared Molecular Genetic Mechanisms. Schizophr Bull.

[13] Roelfs D et al. Genetic overlap between multivariate measures of human functional brain connectivity and psychiatric disorders. *medRxiv* 2021.06.15.21258954 (2021) doi:10.1101/2021.06.15.21258954.

[14] Anttila V (2018). Analysis of shared heritability in common disorders of the brain. Science.

[15] Bulik-Sullivan B (2015). An atlas of genetic correlations across human diseases and traits. Nat Genet.

[16] Cross-Disorder Group of the Psychiatric Genomics Consortium (2013). Identification of risk loci with shared effects on five major psychiatric disorders: a genome-wide analysis. The Lancet.

[17] Lee PH (2019). Genomic Relationships, novel loci, and pleiotropic mechanisms across eight Psychiatric Disorders. Cell.

[18] Romero C et al. Exploring the genetic overlap between 12 psychiatric disorders. *medRxiv* 2022.04.12.22273763 (2022) 10.1101/2022.04.12.22273763.

[19] Wardenaar KJ, de Jonge P (2013). Diagnostic heterogeneity in psychiatry: towards an empirical solution. BMC Med.

[20] Widiger TA, Clark LA (2000). Toward DSM—V and the classification of psychopathology. Psychol Bull.

[21] Kessler RC (2005). Lifetime prevalence and age-of-onset distributions of DSM-IV Disorders in the National Comorbidity Survey Replication. Arch Gen Psychiatry.

[22] McGrath JJ (2015). Psychotic Experiences in the General Population: a cross-national analysis based on 31,261 respondents from 18 countries. JAMA Psychiatry.

[23] Doherty JL, Owen MJ (2014). Genomic insights into the overlap between psychiatric disorders: implications for research and clinical practice. Genome Med.

[24] Roelfs D (2021). Phenotypically independent profiles relevant to mental health are genetically correlated. Transl Psychiatry.

[25] van der Meer D (2020). Understanding the genetic determinants of the brain with MOSTest. Nat Commun.

[26] Sudlow C (2015). UK biobank: an open access resource for identifying the causes of a wide range of complex diseases of middle and old age. PLoS Med.

[27] Alfaro-Almagro F (2018). Image processing and Quality Control for the first 10,000 brain imaging datasets from UK Biobank. NeuroImage.

[28] Bycroft C (2018). The UK Biobank resource with deep phenotyping and genomic data. Nature.

[29] Watanabe K, Taskesen E, van Bochoven A, Posthuma D (2017). Functional mapping and annotation of genetic associations with FUMA. Nat Commun.

[30] de Leeuw CA, Mooij JM, Heskes T, Posthuma DMAGMA (2015). Generalized gene-set analysis of GWAS Data. PLOS Comput Biol.

[31] Jassal B (2020). The reactome pathway knowledgebase. Nucleic Acids Res.

[32] Andreassen OA (2013). Improved detection of common variants associated with schizophrenia and bipolar disorder using pleiotropy-informed conditional false discovery rate. PLoS Genet.

[33] Demontis D (2019). Discovery of the first genome-wide significant risk loci for attention deficit/hyperactivity disorder. Nat Genet.

[34] Grove J (2019). Identification of common genetic risk variants for autism spectrum disorder. Nat Genet.

[35] Duncan LE (2018). Largest GWAS of PTSD (N = 20 070) yields genetic overlap with schizophrenia and sex differences in heritability. Mol Psychiatry.

[36] Smeland OB (2020). Discovery of shared genomic loci using the conditional false discovery rate approach. Hum Genet.

[37] Visscher PM (2017). 10 years of GWAS Discovery: Biology, function, and translation. Am J Hum Genet.

[38] Devor A (2017). Genetic evidence for role of integration of fast and slow neurotransmission in schizophrenia. Mol Psychiatry.

[39] Hsu W-CJ, Nilsson CL, Laezza F (2014). Role of the axonal initial segment in psychiatric disorders: function, dysfunction, and intervention. Front Psychiatry.

[40] Mukai J (2015). Molecular substrates of altered axonal growth and brain connectivity in a mouse model of schizophrenia. Neuron.

[41] Dutt RK (2022). Mental health in the UK Biobank: a roadmap to self-report measures and neuroimaging correlates. Hum Brain Mapp.

[42] Lyall DM (2022). Quantifying bias in psychological and physical health in the UK Biobank imaging sub-sample. Brain Commun.

