# Supplementary material for: Shared genetic architecture between mental health and the brain functional connectome in the UK Biobank
Source: BMC Psychiatry. 2023 Jun 23;23:461. doi: 10.1186/s12888-023-04905-7 (PMC10290393; doi:10.1186/s12888-023-04905-7)
Supplement: Supplementary file 1 — Supplementary Material 1: Supplementary Figures and Tables [file 12888_2023_4905_MOESM1_ESM.docx]

**Supplementary Figures**

| *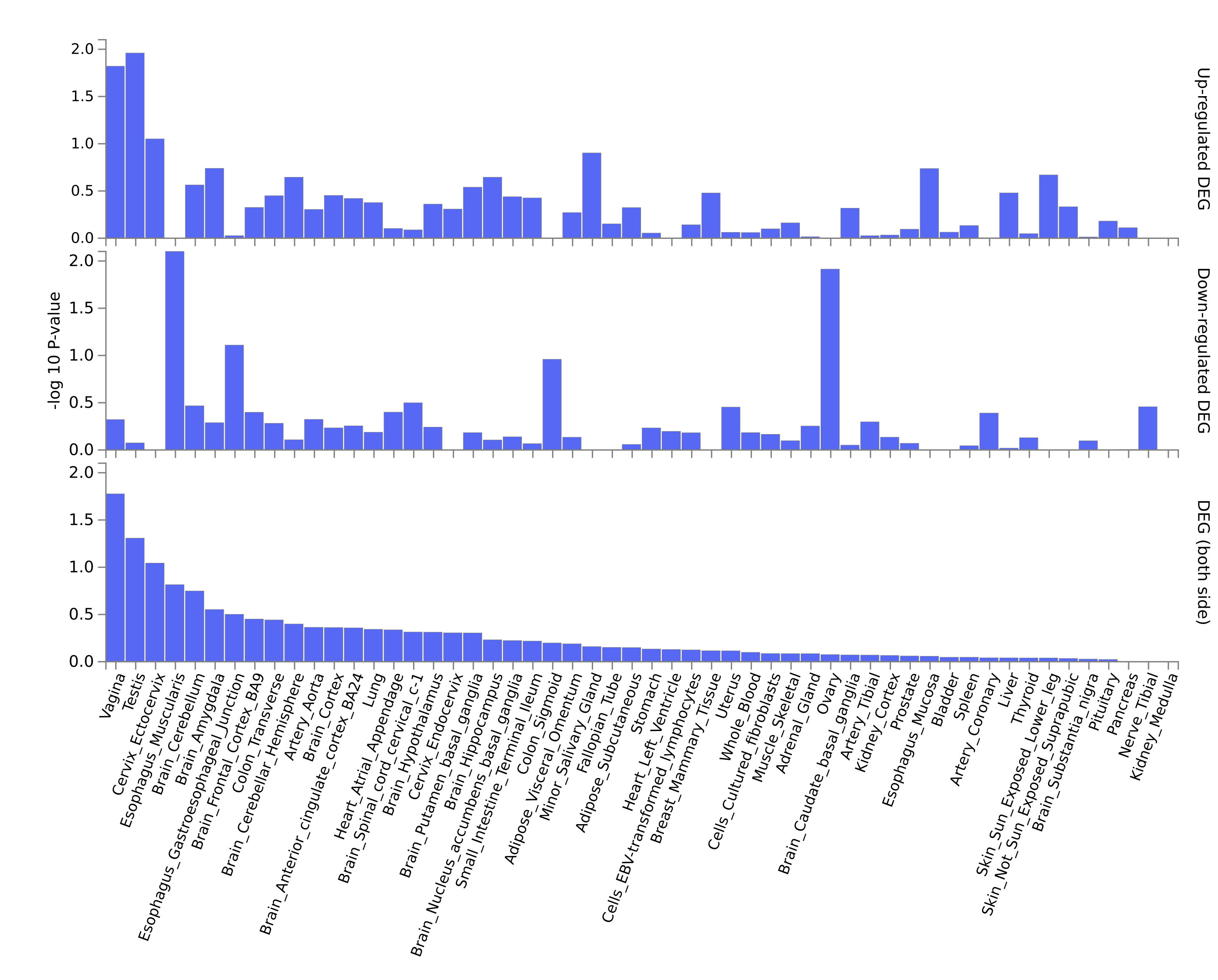* |
| --- |
| **Suppl. Figure 1.** **MAGMA output** List of biological structures mapped by MAGMA implemented through FUMA. Separate facets for up-regulated (top), down-regulated (middle), and both directions (bottom). Among some tangentially related structures (e.g. sexual organs, esophagus), there are large number of items on the higher end of the spectrum are related to brain structures (e.g. cerebellum, amygdala, cortex). |

| *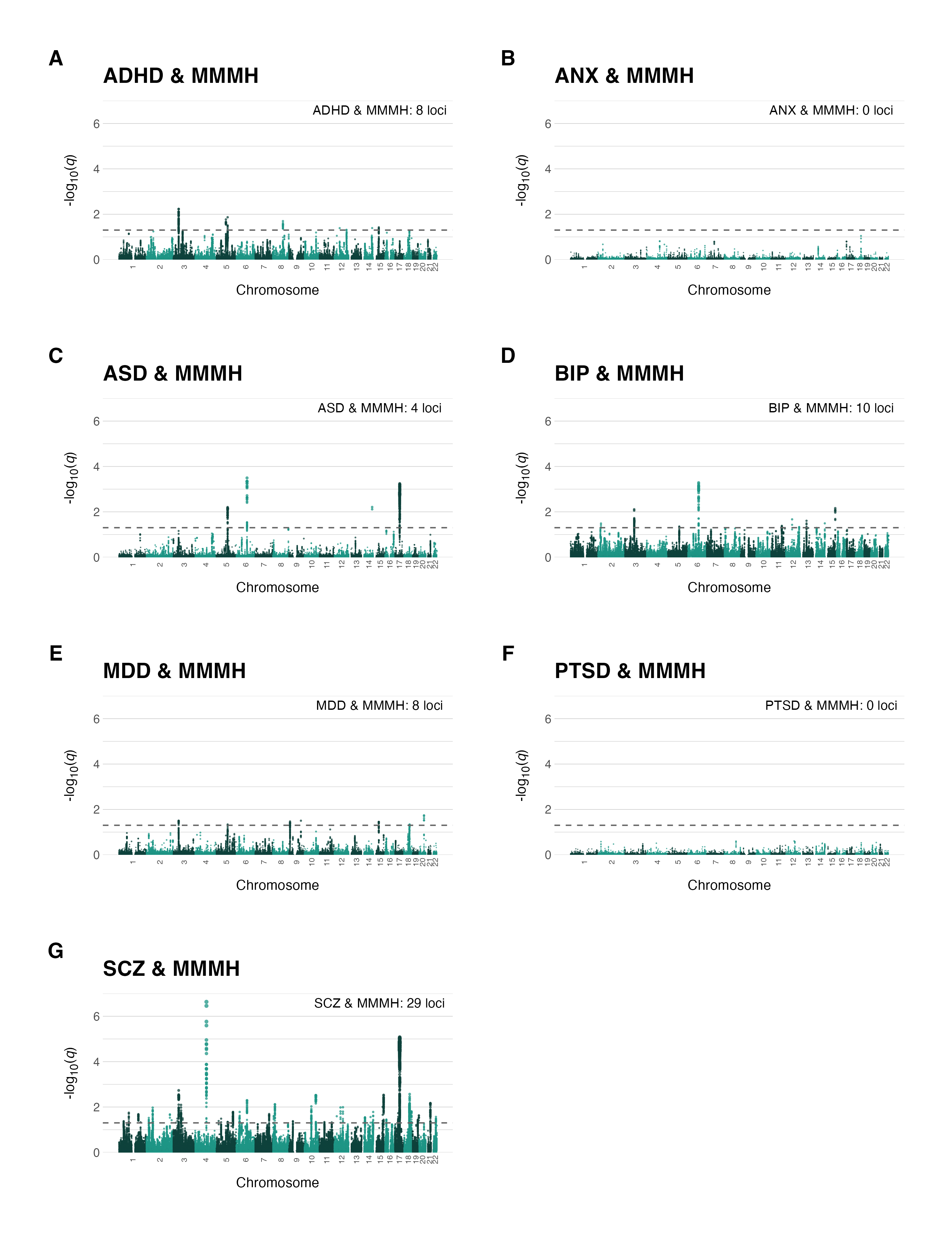* |
| --- |
| **Suppl. Figure 2. Manhattan plots from conjFDR between psychiatric disorders and the multivariate genome-wide association statistics for mental health** Manhattan plots illustrating the shared genetic determinants between a number of psychiatric disorders and the multivariate genome-wide association statistics for mental health. It shows the largest number of shared loci between the multivariate summary statistics and SCZ (29 loci), BIP (10 loci), followed by ADHD and MDD (both 8 loci). |

| *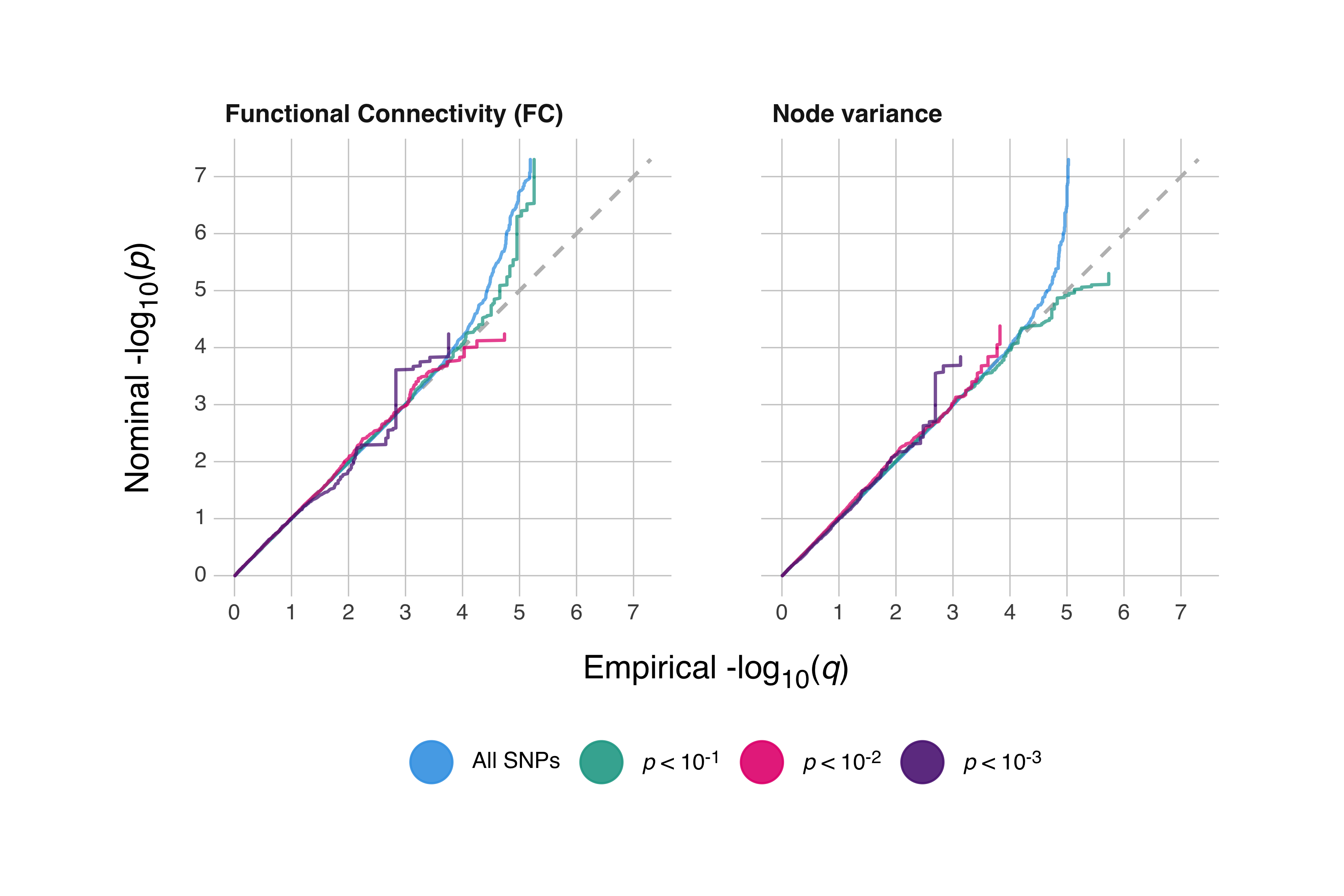* |
| --- |
| **Suppl. Figure 3.** **QQ plots of the genetic signal of the conjFDR between the multivariate genome-wide association statistics for mental health and the brain functional connectome** Figure showing no inflation of signal in the conjFDR at different thresholds. Genetic signal overall was not very strong, but sufficient for the analyses in this pipeline. |

**Supplementary Tables**

| **Suppl. Table 1. Mapped genes from the multivariate genome-wide association statistics for mental health** Genes mapped by FUMA on the summary statistics from the multivariate summary statistics. We found in total 48 genes associated with the genetic signal of the multivariate measure. | | | | |
| --- | --- | --- | --- | --- |
| *No* | *Gene* | *Chr* | *Pmin* | *Ind. Sig. SNP* |
| 1 | CRB1 | 1 | 2.128e-07 | rs17641524 |
| 2 | DENND1B | 1 | 1.060e-08 | rs17641524 |
| 3 | BSN | 3 | 2.759e-04 | rs3749237 |
| 4 | MST1 | 3 | 2.871e-08 | rs3749237 |
| 5 | RNF123 | 3 | 2.871e-08 | rs3749237 |
| 6 | AMIGO3 | 3 | 1.213e-08 | rs3749237 |
| 7 | GMPPB | 3 | 1.213e-08 | rs3749237 |
| 8 | IP6K1 | 3 | 1.213e-08 | rs3749237 |
| 9 | CDHR4 | 3 | 2.898e-08 | rs3749237 |
| 10 | FAM212A | 3 | 4.738e-08 | rs3749237 |
| 11 | UBA7 | 3 | 4.738e-08 | rs3749237 |
| 12 | TRAIP | 3 | 2.802e-08 | rs3749237, rs4688686 |
| 13 | CAMKV | 3 | 2.802e-08 | rs3749237, rs4688686 |
| 14 | MST1R | 3 | 6.717e-08 | rs4688686 |
| 15 | CTD-2330K9.3 | 3 | 6.717e-08 | rs4688686 |
| 16 | MON1A | 3 | 1.294e-07 | rs4688686 |
| 17 | RBM6 | 3 | 8.472e-08 | rs4688686 |
| 18 | RBM5 | 3 | 6.544e-08 | rs4688686 |
| 19 | SEMA3F | 3 | 5.478e-07 | rs4688686 |
| 20 | KLB | 4 | 4.007e-10 | rs35538052 |
| 21 | METAP1 | 4 | 2.364e-08 | rs146788033 |
| 22 | ADH5 | 4 | 2.680e-19 | rs551676206 |
| 23 | ADH4 | 4 | 3.073e-03 | rs146788033 |
| 24 | ADH1B | 4 | 5.678e-41 | rs1229984, rs551676206 |
| 25 | C4orf17 | 4 |  | rs551676206 |
| 26 | TRMT10A | 4 |  | rs551676206 |
| 27 | BANK1 | 4 | 5.882e-09 | rs13107325 |
| 28 | SLC39A8 | 4 | 7.204e-11 | rs13107325, rs13105581 |
| 29 | IRF4 | 6 | 2.298e-08 | rs12203592 |
| 30 | PRRX2 | 9 | 2.093e-09 | rs28428946 |
| 31 | DCAF5 | 14 | 2.136e-07 | rs35538328 |
| 32 | EXD2 | 14 | 2.447e-06 | rs35538328 |
| 33 | ARHGAP27 | 17 | 1.158e-09 | rs62055718 |
| 34 | PLEKHM1 | 17 | 1.534e-13 | rs62055718 |
| 35 | CRHR1 | 17 | 2.897e-14 | rs62055718, rs199440, rs55938136, rs7224296, rs12938031 |
| 36 | SPPL2C | 17 | 4.195e-13 | rs62055718, rs199440, rs7224296, rs12938031 |
| 37 | MAPT | 17 | 1.256e-13 | rs62055718, rs199440, rs7224296 |
| 38 | STH | 17 | 1.437e-13 | rs62055718, rs199440, rs7224296 |
| 39 | KANSL1 | 17 | 7.431e-14 | rs62055718, rs199440, rs7224296 |
| 40 | ARL17B | 17 | 3.191e-14 | rs62055718, rs199440, rs7224296 |
| 41 | LRRC37A | 17 | 5.397e-13 | rs62055718, rs199440, rs7224296 |
| 42 | LRRC37A2 | 17 |  | rs62055718, rs199440, rs7224296 |
| 43 | ARL17A | 17 |  | rs62055718, rs199440, rs7224296 |
| 44 | NSF | 17 | 3.552e-12 | rs62055718, rs199440, rs7224296, rs199470 |
| 45 | WNT3 | 17 | 7.193e-12 | rs199440, rs7224296 |
| 46 | PSMG1 | 21 | 3.415e-07 | rs7281463 |
| 47 | BRWD1 | 21 | 2.851e-07 | rs7281463 |
| 48 | HMGN1 | 21 | 2.425e-06 | rs7281463 |

| **Suppl. Table 2. Overview of the genetic association analyses on psychiatric conditions used in this study** List of GWASs we included in the genetic analyses in this paper and their meta data. Sample size in the table and in the summary statistics used in the final analyses may differ since we excluded individuals already included in the UK Biobank and included only individuals with White European ancestry. | | | | | | | | |
| --- | --- | --- | --- | --- | --- | --- | --- | --- |
| *Pheno-*  *type* | *Consortium* | *Sample* | *Citation* | *N_case_* | *N_control_* | *Number of loci* | *LambdaGC* | *h2*  *(se)* |
| SCZ | PGC | Meta analysis of European individuals in PGC and CLOZUK samples | Pardiñas et al., 2018 | 40,675 | 64,643 | 135 | 1.6831 | 0.41 (0.01) |
| BIP | PGC | European individuals | Mullins et al., 2021 | 41,917 | 371,549 | 44 | 1.4316 | 0.0853 (<0.01) |
| MDD | PGC | European individuals | Wray et al., 2018 | 69,576 | 161,613 | 2 | 1.1973 | 0.0717 (<0.01) |
| ADHD | PGC | European individuals | Demontis et al., 2019 | 19,099 | 34,194 | 12 | 1.2531 | 0.2354 (0.02) |
| ASD | PGC, iPSYCH | Meta analysis of European individuals | Grove et al., 2019 | 18,381 | 27,969 | 2 | 1.1715 | 0.1941 (0.02) |
| PTSD | PGC | European individuals | Duncan et al., 2018 | 2,424 | 7,113 | 0 | 1.0165 | 0.101 (0.04) |
| ANX | ANGST | Meta analysis of European individuals | Otowa et al., 2016 | 7,016 | 14,745 | 1 | 1.0345 | 0.0763 (0.03) |

| **Suppl. Table 3. Number of overlapping genes in gene sets with the multivariate genome-wide association statistics for mental health (MMMH)** Table showing the number of overlapping genes identified through either overlap in gene sets, the number of genes from the disorder GWAS alone, and the number of shared genes identified through conjFDR. | | | | |
| --- | --- | --- | --- | --- |
| *Disorder* | *Measure* | *Number of overlapping genes in gene sets* | *Number of genes in disorder GWAS alone* | *Number of shared genes identified through cFDR* |
| ADHD | MMMH | 1 | 22 | 37 |
| ANX | MMMH | 0 | 0 | 0 |
| ASD | MMMH | 0 | 6 | 16 |
| BIP | MMMH | 7 | 184 | 28 |
| MDD | MMMH | 0 | 1 | 29 |
| PTSD | MMMH | 0 | 0 | 0 |
| SCZ | MMMH | 27 | 600 | 72 |

| **Suppl. Table 4. List of genes associated with the shared genetic determinants between the psychiatric disorders and multivariate genome-wide association statistics for mental health** Genes associated with conjFDR output from diagnosis and multivariate genome-wide association statistics for mental health. This list only includes psychiatric disorders for which we identified significant loci. The number of mapped genes can be greater than the number of discovered loci since loci can be associated with more than one gene. | | | | | | |
| --- | --- | --- | --- | --- | --- | --- |
| *Diagnosis* | *Measure* | *No* | *Gene* | *Chr* | *Pmin* | *Ind. Sig. SNP* |
| SCZ | MMMH | 1 | BARHL2 | 1 | 2.568e-07 | rs12046000 |
| SCZ | MMMH | 2 | CRB1 | 1 | 4.704e-07 | rs10922218 |
| SCZ | MMMH | 3 | DENND1B | 1 | 1.907e-06 | rs10922218 |
| SCZ | MMMH | 4 | FANCL | 2 | 1.892e-06 | rs2125924 |
| SCZ | MMMH | 5 | BCL11A | 2 | 4.375e-07 | rs13019832 |
| SCZ | MMMH | 6 | XYLB | 3 | 3.220e-07 | rs151618 |
| SCZ | MMMH | 7 | EXOG | 3 | 3.220e-07 | rs151618 |
| SCZ | MMMH | 8 | CCDC71 | 3 | 1.795e-07 | rs2624847 |
| SCZ | MMMH | 9 | GPX1 | 3 | 1.795e-07 | rs2624847 |
| SCZ | MMMH | 10 | AMT | 3 | 1.795e-07 | rs2624847 |
| SCZ | MMMH | 11 | FAM212A | 3 | 1.795e-07 | rs2624847 |
| SCZ | MMMH | 12 | MST1R | 3 | 1.795e-07 | rs2624847 |
| SCZ | MMMH | 13 | RBM6 | 3 | 1.795e-07 | rs2624847 |
| SCZ | MMMH | 14 | FOXP1 | 3 | 2.086e-07 | rs60756930 |
| SCZ | MMMH | 15 | BANK1 | 4 | 1.651e-10 | rs13107325 |
| SCZ | MMMH | 16 | SLC39A8 | 4 | 3.472e-12 | rs13107325 |
| SCZ | MMMH | 17 | HCN1 | 5 | 1.601e-06 | rs10941692 |
| SCZ | MMMH | 18 | ZCCHC7 | 9 | 4.373e-07 | rs308523 |
| SCZ | MMMH | 19 | RTKN2 | 10 | 2.944e-07 | rs2306970 |
| SCZ | MMMH | 20 | ZNF365 | 10 | 9.392e-08 | rs2306970 |
| SCZ | MMMH | 21 | TMEM180 | 10 | 1.628e-06 | rs7909286 |
| SCZ | MMMH | 22 | ARL3 | 10 | 4.597e-08 | rs7909286 |
| SCZ | MMMH | 23 | C10orf32 | 10 | 6.281e-07 | rs7909286 |
| SCZ | MMMH | 24 | C10orf32-ASMT | 10 | 4.597e-08 | rs7909286 |
| SCZ | MMMH | 25 | AS3MT | 10 | 4.597e-08 | rs7909286 |
| SCZ | MMMH | 26 | CNNM2 | 10 | 4.597e-08 | rs7909286 |
| SCZ | MMMH | 27 | NT5C2 | 10 | 6.005e-06 | rs7909286 |
| SCZ | MMMH | 28 | TAC3 | 12 | 1.549e-07 | rs703848 |
| SCZ | MMMH | 29 | MYO1A | 12 | 1.549e-07 | rs703848 |
| SCZ | MMMH | 30 | TMEM194A | 12 | 2.860e-07 | rs703848 |
| SCZ | MMMH | 31 | NAB2 | 12 | 2.860e-07 | rs703848 |
| SCZ | MMMH | 32 | STAT6 | 12 | 2.860e-07 | rs703848 |
| SCZ | MMMH | 33 | SYT1 | 12 | 1.021e-07 | rs6539344 |
| SCZ | MMMH | 34 | PRKD1 | 14 | 3.714e-07 | rs10134319 |
| SCZ | MMMH | 35 | NRXN3 | 14 | 2.761e-07 | rs17764956 |
| SCZ | MMMH | 36 | APOPT1 | 14 | 2.421e-07 | rs2295146 |
| SCZ | MMMH | 37 | XRCC3 | 14 | 1.022e-06 | rs2295146 |
| SCZ | MMMH | 38 | AL049840.1 | 14 | 3.317e-06 | rs2295146 |
| SCZ | MMMH | 39 | ZFYVE21 | 14 | 2.421e-07 | rs2295146 |
| SCZ | MMMH | 40 | PPP1R13B | 14 | 2.421e-07 | rs2295146 |
| SCZ | MMMH | 41 | KIF26A | 14 | 3.317e-06 | rs2295146 |
| SCZ | MMMH | 42 | GOLGA6L9 | 15 | 9.837e-08 | rs4778687 |
| SCZ | MMMH | 43 | CPEB1 | 15 | 9.837e-08 | rs4778687 |
| SCZ | MMMH | 44 | AP3B2 | 15 | 9.837e-08 | rs4778687 |
| SCZ | MMMH | 45 | FURIN | 15 | 4.469e-08 | rs4932178 |
| SCZ | MMMH | 46 | FES | 15 | 5.858e-08 | rs4932178 |
| SCZ | MMMH | 47 | MAN2A2 | 15 |  | rs4932178 |
| SCZ | MMMH | 48 | RCCD1 | 15 | 7.716e-08 | rs4932178 |
| SCZ | MMMH | 49 | RHBDL3 | 17 | 4.403e-07 | rs9916114 |
| SCZ | MMMH | 50 | C17orf75 | 17 | 9.641e-07 | rs9916114 |
| SCZ | MMMH | 51 | ZNF207 | 17 | 7.052e-07 | rs9916114 |
| SCZ | MMMH | 52 | PSMD11 | 17 | 4.403e-07 | rs9916114 |
| SCZ | MMMH | 53 | CDK5R1 | 17 | 5.273e-07 | rs9916114 |
| SCZ | MMMH | 54 | MYO1D | 17 | 5.273e-07 | rs9916114 |
| SCZ | MMMH | 55 | NMT1 | 17 | 6.944e-08 | rs2532392 |
| SCZ | MMMH | 56 | FMNL1 | 17 | 5.389e-08 | rs2532392 |
| SCZ | MMMH | 57 | ARHGAP27 | 17 | 5.389e-08 | rs2532392 |
| SCZ | MMMH | 58 | PLEKHM1 | 17 | 5.389e-08 | rs2532392 |
| SCZ | MMMH | 59 | CRHR1 | 17 | 5.389e-08 | rs2532392 |
| SCZ | MMMH | 60 | SPPL2C | 17 | 5.389e-08 | rs2532392 |
| SCZ | MMMH | 61 | MAPT | 17 | 5.389e-08 | rs2532392 |
| SCZ | MMMH | 62 | STH | 17 | 1.145e-07 | rs2532392 |
| SCZ | MMMH | 63 | KANSL1 | 17 | 5.090e-08 | rs2532392 |
| SCZ | MMMH | 64 | ARL17B | 17 | 6.864e-08 | rs2532392 |
| SCZ | MMMH | 65 | LRRC37A | 17 | 5.389e-08 | rs2532392 |
| SCZ | MMMH | 66 | LRRC37A2 | 17 | 5.389e-08 | rs2532392 |
| SCZ | MMMH | 67 | ARL17A | 17 | 5.389e-08 | rs2532392 |
| SCZ | MMMH | 68 | NSF | 17 | 5.389e-08 | rs2532392 |
| SCZ | MMMH | 69 | WNT3 | 17 | 1.057e-07 | rs2532392 |
| SCZ | MMMH | 70 | EFCAB13 | 17 | 5.879e-07 | rs2532392 |
| SCZ | MMMH | 71 | DCC | 18 | 1.048e-07 | rs10502971 |
| SCZ | MMMH | 72 | TCF4 | 18 | 4.085e-08 | rs7240986 |
| MDD | MMMH | 1 | NCKIPSD | 3 | 3.142e-07 | rs2029591 |
| MDD | MMMH | 2 | P4HTM | 3 | 3.142e-07 | rs2029591 |
| MDD | MMMH | 3 | WDR6 | 3 | 3.142e-07 | rs2029591 |
| MDD | MMMH | 4 | DALRD3 | 3 | 3.142e-07 | rs2029591 |
| MDD | MMMH | 5 | QRICH1 | 3 | 3.142e-07 | rs2029591 |
| MDD | MMMH | 6 | CCDC71 | 3 | 3.142e-07 | rs2029591 |
| MDD | MMMH | 7 | GPX1 | 3 | 3.142e-07 | rs2029591 |
| MDD | MMMH | 8 | RHOA | 3 |  | rs2029591 |
| MDD | MMMH | 9 | AMT | 3 | 3.142e-07 | rs2029591 |
| MDD | MMMH | 10 | BSN | 3 | 3.142e-07 | rs2029591 |
| MDD | MMMH | 11 | APEH | 3 | 1.386e-06 | rs2029591 |
| MDD | MMMH | 12 | MST1 | 3 | 1.386e-06 | rs2029591 |
| MDD | MMMH | 13 | RNF123 | 3 | 3.142e-07 | rs2029591 |
| MDD | MMMH | 14 | AMIGO3 | 3 | 1.088e-06 | rs2029591 |
| MDD | MMMH | 15 | GMPPB | 3 | 3.142e-07 | rs2029591 |
| MDD | MMMH | 16 | IP6K1 | 3 | 8.869e-07 | rs2029591 |
| MDD | MMMH | 17 | CDHR4 | 3 | 1.215e-06 | rs2029591 |
| MDD | MMMH | 18 | FAM212A | 3 | 8.869e-07 | rs2029591 |
| MDD | MMMH | 19 | UBA7 | 3 | 1.532e-06 | rs2029591 |
| MDD | MMMH | 20 | TRAIP | 3 | 1.290e-06 | rs2029591 |
| MDD | MMMH | 21 | CAMKV | 3 | 1.487e-06 | rs2029591 |
| MDD | MMMH | 22 | RBM6 | 3 | 4.871e-07 | rs2029591 |
| MDD | MMMH | 23 | HYAL3 | 3 | 3.142e-07 | rs2029591 |
| MDD | MMMH | 24 | ZNF462 | 9 | 3.141e-07 | rs11789013 |
| MDD | MMMH | 25 | RP11-508N12.4 | 9 | 3.141e-07 | rs11789013 |
| MDD | MMMH | 26 | SEMA6D | 15 | 3.546e-07 | rs4338738 |
| MDD | MMMH | 27 | DCC | 18 | 4.743e-07 | rs8084351 |
| MDD | MMMH | 28 | TCF4 | 18 | 4.594e-07 | rs2924336 |
| MDD | MMMH | 29 | ZMYND8 | 20 | 1.821e-07 | rs6094628 |
| BIP | MMMH | 1 | TRIM54 | 2 | 4.702e-07 | rs1260326 |
| BIP | MMMH | 2 | EIF2B4 | 2 | 3.833e-07 | rs1260326 |
| BIP | MMMH | 3 | SNX17 | 2 | 3.833e-07 | rs1260326 |
| BIP | MMMH | 4 | ZNF513 | 2 | 3.833e-07 | rs1260326 |
| BIP | MMMH | 5 | PPM1G | 2 | 3.833e-07 | rs1260326 |
| BIP | MMMH | 6 | KRTCAP3 | 2 | 3.253e-07 | rs1260326 |
| BIP | MMMH | 7 | GCKR | 2 | 3.253e-07 | rs1260326 |
| BIP | MMMH | 8 | AC109829.1 | 2 | 1.823e-06 | rs1260326 |
| BIP | MMMH | 9 | CADM2 | 3 | 7.779e-08 | rs1872552 |
| BIP | MMMH | 10 | CNTN5 | 11 | 4.121e-07 | rs671129 |
| BIP | MMMH | 11 | TAC3 | 12 | 2.144e-07 | rs703848 |
| BIP | MMMH | 12 | MYO1A | 12 | 2.144e-07 | rs703848 |
| BIP | MMMH | 13 | TMEM194A | 12 | 3.734e-07 | rs703848 |
| BIP | MMMH | 14 | NAB2 | 12 | 3.734e-07 | rs703848 |
| BIP | MMMH | 15 | STAT6 | 12 | 3.734e-07 | rs703848 |
| BIP | MMMH | 16 | CABP1 | 12 | 4.644e-07 | rs56366632 |
| BIP | MMMH | 17 | MLEC | 12 | 1.524e-06 | rs56366632 |
| BIP | MMMH | 18 | UNC119B | 12 | 1.373e-06 | rs56366632 |
| BIP | MMMH | 19 | ACADS | 12 | 1.060e-06 | rs56366632 |
| BIP | MMMH | 20 | SPPL3 | 12 | 2.124e-06 | rs56366632 |
| BIP | MMMH | 21 | APOPT1 | 14 | 3.221e-07 | rs2295146 |
| BIP | MMMH | 22 | XRCC3 | 14 | 1.311e-06 | rs2295146 |
| BIP | MMMH | 23 | AL049840.1 | 14 | 3.618e-06 | rs2295146 |
| BIP | MMMH | 24 | ZFYVE21 | 14 | 3.221e-07 | rs2295146 |
| BIP | MMMH | 25 | PPP1R13B | 14 | 3.221e-07 | rs2295146 |
| BIP | MMMH | 26 | KIF26A | 14 | 3.618e-06 | rs2295146 |
| BIP | MMMH | 27 | FURIN | 15 | 6.946e-08 | rs1894401 |
| BIP | MMMH | 28 | FES | 15 | 6.946e-08 | rs1894401 |
| ASD | MMMH | 1 | SERPINA1 | 14 | 6.175e-08 | rs28929474 |
| ASD | MMMH | 2 | NMT1 | 17 | 7.554e-09 | rs71375338 |
| ASD | MMMH | 3 | FMNL1 | 17 | 6.107e-09 | rs71375338 |
| ASD | MMMH | 4 | ARHGAP27 | 17 | 5.686e-09 | rs71375338 |
| ASD | MMMH | 5 | PLEKHM1 | 17 | 5.686e-09 | rs71375338 |
| ASD | MMMH | 6 | CRHR1 | 17 | 6.107e-09 | rs71375338 |
| ASD | MMMH | 7 | SPPL2C | 17 | 5.686e-09 | rs71375338 |
| ASD | MMMH | 8 | MAPT | 17 | 6.107e-09 | rs71375338 |
| ASD | MMMH | 9 | STH | 17 | 1.158e-08 | rs71375338 |
| ASD | MMMH | 10 | KANSL1 | 17 | 6.107e-09 | rs71375338 |
| ASD | MMMH | 11 | ARL17B | 17 | 6.282e-09 | rs71375338 |
| ASD | MMMH | 12 | LRRC37A | 17 | 5.686e-09 | rs71375338 |
| ASD | MMMH | 13 | LRRC37A2 | 17 | 5.686e-09 | rs71375338 |
| ASD | MMMH | 14 | ARL17A | 17 | 5.686e-09 | rs71375338 |
| ASD | MMMH | 15 | NSF | 17 | 6.107e-09 | rs71375338 |
| ASD | MMMH | 16 | WNT3 | 17 | 1.160e-08 | rs71375338 |
| ADHD | MMMH | 1 | NCKIPSD | 3 | 1.069e-07 | rs373797031 |
| ADHD | MMMH | 2 | P4HTM | 3 | 1.069e-07 | rs373797031 |
| ADHD | MMMH | 3 | WDR6 | 3 | 1.069e-07 | rs373797031 |
| ADHD | MMMH | 4 | DALRD3 | 3 | 1.069e-07 | rs373797031 |
| ADHD | MMMH | 5 | QRICH1 | 3 | 1.102e-07 | rs373797031 |
| ADHD | MMMH | 6 | CCDC71 | 3 | 1.069e-07 | rs373797031 |
| ADHD | MMMH | 7 | GPX1 | 3 | 1.069e-07 | rs373797031 |
| ADHD | MMMH | 8 | AMT | 3 | 1.069e-07 | rs373797031 |
| ADHD | MMMH | 9 | BSN | 3 | 1.069e-07 | rs373797031 |
| ADHD | MMMH | 10 | APEH | 3 | 5.050e-07 | rs373797031 |
| ADHD | MMMH | 11 | MST1 | 3 | 5.050e-07 | rs373797031 |
| ADHD | MMMH | 12 | RNF123 | 3 | 1.069e-07 | rs373797031 |
| ADHD | MMMH | 13 | AMIGO3 | 3 | 5.137e-07 | rs373797031 |
| ADHD | MMMH | 14 | GMPPB | 3 | 1.069e-07 | rs373797031 |
| ADHD | MMMH | 15 | IP6K1 | 3 | 5.901e-08 | rs373797031 |
| ADHD | MMMH | 16 | CDHR4 | 3 | 4.367e-07 | rs373797031 |
| ADHD | MMMH | 17 | FAM212A | 3 | 2.557e-07 | rs373797031 |
| ADHD | MMMH | 18 | UBA7 | 3 | 4.367e-07 | rs373797031 |
| ADHD | MMMH | 19 | TRAIP | 3 | 5.619e-07 | rs373797031 |
| ADHD | MMMH | 20 | CAMKV | 3 | 9.356e-07 | rs373797031 |
| ADHD | MMMH | 21 | RBM6 | 3 | 1.998e-07 | rs373797031 |
| ADHD | MMMH | 22 | HYAL3 | 3 | 1.069e-07 | rs373797031 |
| ADHD | MMMH | 23 | MEF2C | 5 | 1.695e-07 | rs304136 |
| ADHD | MMMH | 24 | SPATS2 | 12 | 8.567e-06 | rs933738 |
| ADHD | MMMH | 25 | KCNH3 | 12 | 4.095e-07 | rs933738 |
| ADHD | MMMH | 26 | MCRS1 | 12 | 4.095e-07 | rs933738 |
| ADHD | MMMH | 27 | PRPF40B | 12 | 1.716e-06 | rs933738 |
| ADHD | MMMH | 28 | FAM186B | 12 | 1.716e-06 | rs933738 |
| ADHD | MMMH | 29 | FMNL3 | 12 | 7.508e-06 | rs933738 |
| ADHD | MMMH | 30 | TMBIM6 | 12 | 7.091e-06 | rs933738 |
| ADHD | MMMH | 31 | SH2B3 | 12 | 8.013e-07 | rs10849943 |
| ADHD | MMMH | 32 | ATXN2 | 12 | 3.011e-06 | rs10849943 |
| ADHD | MMMH | 33 | ALDH2 | 12 | 4.842e-07 | rs10849943 |
| ADHD | MMMH | 34 | TMEM116 | 12 | 9.193e-07 | rs10849943 |
| ADHD | MMMH | 35 | NAA25 | 12 | 4.842e-07 | rs10849943 |
| ADHD | MMMH | 36 | SERPINA1 | 14 | 4.065e-07 | rs112635299 |
| ADHD | MMMH | 37 | SEMA6D | 15 | 3.759e-07 | rs281287 |

| **Suppl. Table 5. Gene list of conjFDR between the brain functional connectome and multivariate genome-wide association statistics for mental health** Genes identified through FUMA associated with the shared loci between the multivariate genome-wide association statistics for mental health and FC or node variance. The number of genes in this gene set can be larger than the number of loci since a locus can be associated more than one gene. | | | | | | |
| --- | --- | --- | --- | --- | --- | --- |
| *Feature* | *Measure* | *No* | *Gene* | *Chr* | *Pmin* | *Ind. Sig. SNP* |
| FC | MMMH | 1 | LRPPRC | 2 | 3.806e-06 | rs62132319 |
| FC | MMMH | 2 | CAMKMT | 2 | 3.807e-07 | rs62132319 |
| FC | MMMH | 3 | FANCL | 2 | 1.683e-06 | rs2125924 |
| FC | MMMH | 4 | BCL11A | 2 | 1.977e-07 | rs13019832 |
| FC | MMMH | 5 | ARL8B | 3 | 4.172e-07 | rs74677825 |
| FC | MMMH | 6 | ZNF589 | 3 | 1.032e-06 | rs6769821 |
| FC | MMMH | 7 | TMA7 | 3 | 4.737e-07 | rs6769821 |
| FC | MMMH | 8 | ATRIP | 3 | 1.134e-06 | rs6769821 |
| FC | MMMH | 9 | TREX1 | 3 | 4.737e-07 | rs6769821 |
| FC | MMMH | 10 | NCKIPSD | 3 | 4.737e-07 | rs6769821 |
| FC | MMMH | 11 | IP6K2 | 3 | 1.738e-06 | rs6769821 |
| FC | MMMH | 12 | PRKAR2A | 3 | 4.737e-07 | rs6769821 |
| FC | MMMH | 13 | SLC25A20 | 3 | 1.499e-06 | rs6769821 |
| FC | MMMH | 14 | ARIH2OS | 3 | 1.532e-06 | rs6769821 |
| FC | MMMH | 15 | ARIH2 | 3 | 1.303e-06 | rs6769821 |
| FC | MMMH | 16 | P4HTM | 3 | 4.737e-07 | rs6769821 |
| FC | MMMH | 17 | WDR6 | 3 | 4.737e-07 | rs6769821 |
| FC | MMMH | 18 | DALRD3 | 3 | 4.737e-07 | rs6769821 |
| FC | MMMH | 19 | NDUFAF3 | 3 | 1.205e-06 | rs6769821 |
| FC | MMMH | 20 | IMPDH2 | 3 | 1.205e-06 | rs6769821 |
| FC | MMMH | 21 | QRICH1 | 3 | 4.737e-07 | rs6769821 |
| FC | MMMH | 22 | QARS | 3 | 8.188e-07 | rs6769821 |
| FC | MMMH | 23 | USP19 | 3 | 8.188e-07 | rs6769821 |
| FC | MMMH | 24 | LAMB2 | 3 | 9.061e-07 | rs6769821 |
| FC | MMMH | 25 | CCDC71 | 3 | 9.878e-08 | rs6769821; rs2624847 |
| FC | MMMH | 26 | KLHDC8B | 3 | 4.898e-07 | rs6769821 |
| FC | MMMH | 27 | C3orf84 | 3 | 4.898e-07 | rs6769821 |
| FC | MMMH | 28 | CCDC36 | 3 | 4.737e-07 | rs6769821 |
| FC | MMMH | 29 | RP11-3B7.1 | 3 | 7.060e-07 | rs6769821 |
| FC | MMMH | 30 | C3orf62 | 3 | 7.060e-07 | rs6769821 |
| FC | MMMH | 31 | USP4 | 3 | 4.737e-07 | rs6769821 |
| FC | MMMH | 32 | GPX1 | 3 | 9.878e-08 | rs6769821; rs2624847 |
| FC | MMMH | 33 | RHOA | 3 | 1.320e-06 | rs6769821 |
| FC | MMMH | 34 | TCTA | 3 | 1.339e-06 | rs6769821 |
| FC | MMMH | 35 | AMT | 3 | 9.878e-08 | rs6769821; rs2624847 |
| FC | MMMH | 36 | NICN1 | 3 | 4.737e-07 | rs6769821 |
| FC | MMMH | 37 | DAG1 | 3 | 1.143e-06 | rs6769821 |
| FC | MMMH | 38 | BSN | 3 | 1.246e-06 | rs6769821 |
| FC | MMMH | 39 | MST1 | 3 | 6.078e-07 | rs6769821 |
| FC | MMMH | 40 | RNF123 | 3 |  | rs6769821 |
| FC | MMMH | 41 | GMPPB | 3 | 4.737e-07 | rs6769821 |
| FC | MMMH | 42 | FAM212A | 3 | 9.878e-08 | rs2624847 |
| FC | MMMH | 43 | MST1R | 3 | 9.878e-08 | rs2624847 |
| FC | MMMH | 44 | RBM6 | 3 | 9.878e-08 | rs2624847 |
| FC | MMMH | 45 | HYAL3 | 3 | 1.039e-06 | rs6769821 |
| FC | MMMH | 46 | CADM2 | 3 | 2.305e-08 | rs4856600 |
| FC | MMMH | 47 | EPHA3 | 3 | 2.806e-07 | rs73153293 |
| FC | MMMH | 48 | BANK1 | 4 | 2.807e-06 | rs13107325 |
| FC | MMMH | 49 | SLC39A8 | 4 | 9.802e-10 | rs13107325 |
| FC | MMMH | 50 | MEF2C | 5 | 2.866e-07 | rs618741 |
| FC | MMMH | 51 | SND1 | 7 | 3.498e-07 | rs58406876 |
| FC | MMMH | 52 | LRRC4 | 7 | 6.509e-07 | rs58406876 |
| FC | MMMH | 53 | ARL3 | 10 | 3.174e-07 | rs12765002 |
| FC | MMMH | 54 | C10orf32 | 10 | 3.174e-07 | rs12765002 |
| FC | MMMH | 55 | C10orf32-ASMT | 10 | 3.174e-07 | rs12765002 |
| FC | MMMH | 56 | AS3MT | 10 | 3.174e-07 | rs12765002 |
| FC | MMMH | 57 | CNNM2 | 10 | 3.174e-07 | rs12765002 |
| FC | MMMH | 58 | NT5C2 | 10 | 3.147e-06 | rs12765002 |
| FC | MMMH | 59 | SH2B3 | 12 | 1.732e-07 | rs12810456 |
| FC | MMMH | 60 | ATXN2 | 12 | 1.035e-07 | rs12810456 |
| FC | MMMH | 61 | BRAP | 12 | 2.655e-06 | rs12810456 |
| FC | MMMH | 62 | ACAD10 | 12 | 2.474e-06 | rs12810456 |
| FC | MMMH | 63 | RP11-162P23.2 | 12 | 2.887e-06 | rs12810456 |
| FC | MMMH | 64 | ALDH2 | 12 | 1.035e-07 | rs12810456 |
| FC | MMMH | 65 | MAPKAPK5 | 12 | 5.899e-07 | rs12810456 |
| FC | MMMH | 66 | TMEM116 | 12 | 1.035e-07 | rs12810456 |
| FC | MMMH | 67 | NAA25 | 12 | 1.035e-07 | rs12810456 |
| FC | MMMH | 68 | NMT1 | 17 | 9.948e-14 | rs56328224; rs55938136 |
| FC | MMMH | 69 | FMNL1 | 17 | 9.948e-14 | rs56328224; rs55938136 |
| FC | MMMH | 70 | ARHGAP27 | 17 | 9.948e-14 | rs56328224; rs55938136 |
| FC | MMMH | 71 | PLEKHM1 | 17 | 9.948e-14 | rs56328224; rs55938136 |
| FC | MMMH | 72 | CRHR1 | 17 | 9.948e-14 | rs56328224; rs55938136 |
| FC | MMMH | 73 | SPPL2C | 17 | 9.948e-14 | rs56328224; rs55938136 |
| FC | MMMH | 74 | MAPT | 17 | 9.948e-14 | rs56328224; rs55938136 |
| FC | MMMH | 75 | STH | 17 | 9.948e-14 | rs56328224 |
| FC | MMMH | 76 | KANSL1 | 17 | 9.948e-14 | rs56328224; rs55938136 |
| FC | MMMH | 77 | ARL17B | 17 | 9.948e-14 | rs56328224 |
| FC | MMMH | 78 | LRRC37A | 17 | 9.948e-14 | rs56328224; rs55938136 |
| FC | MMMH | 79 | LRRC37A2 | 17 | 9.948e-14 | rs56328224; rs55938136 |
| FC | MMMH | 80 | ARL17A | 17 | 9.948e-14 | rs56328224; rs55938136 |
| FC | MMMH | 81 | NSF | 17 | 9.948e-14 | rs56328224; rs55938136 |
| FC | MMMH | 82 | WNT3 | 17 | 9.948e-14 | rs56328224 |
| FC | MMMH | 83 | ATP5G1 | 17 | 4.306e-07 | rs11079849 |
| FC | MMMH | 84 | UBE2Z | 17 | 2.101e-06 | rs11079849 |
| FC | MMMH | 85 | SNF8 | 17 | 4.306e-07 | rs11079849 |
| FC | MMMH | 86 | IGF2BP1 | 17 | 4.306e-07 | rs11079849 |
| FC | MMMH | 87 | NGFR | 17 | 6.727e-06 | rs11079849 |
| node variance | MMMH | 1 | SIX3 | 2 | 4.062e-07 | rs490340 |
| node variance | MMMH | 2 | BANK1 | 4 | 6.972e-08 | rs13109404 |
| node variance | MMMH | 3 | EXOC4 | 7 | 7.507e-08 | rs6467507 |
| node variance | MMMH | 4 | NMT1 | 17 | 4.007e-12 | rs71375338; rs55938136 |
| node variance | MMMH | 5 | FMNL1 | 17 | 4.007e-12 | rs71375338; rs55938136 |
| node variance | MMMH | 6 | ARHGAP27 | 17 | 2.850e-12 | rs71375338; rs55938136 |
| node variance | MMMH | 7 | PLEKHM1 | 17 | 2.850e-12 | rs71375338; rs55938136 |
| node variance | MMMH | 8 | CRHR1 | 17 | 4.007e-12 | rs71375338; rs55938136 |
| node variance | MMMH | 9 | SPPL2C | 17 | 2.850e-12 | rs71375338; rs55938136 |
| node variance | MMMH | 10 | MAPT | 17 | 4.007e-12 | rs71375338; rs55938136 |
| node variance | MMMH | 11 | STH | 17 | 7.119e-12 | rs71375338 |
| node variance | MMMH | 12 | KANSL1 | 17 | 4.007e-12 | rs71375338; rs76527351; rs55938136 |
| node variance | MMMH | 13 | ARL17B | 17 | 3.084e-11 | rs71375338 |
| node variance | MMMH | 14 | LRRC37A | 17 | 2.850e-12 | rs71375338; rs55938136 |
| node variance | MMMH | 15 | LRRC37A2 | 17 | 2.850e-12 | rs71375338; rs55938136 |
| node variance | MMMH | 16 | ARL17A | 17 | 2.850e-12 | rs71375338; rs55938136 |
| node variance | MMMH | 17 | NSF | 17 | 4.405e-12 | rs71375338; rs55938136 |
| node variance | MMMH | 18 | WNT3 | 17 | 1.450e-11 | rs71375338 |

| **Suppl. Table 6. Biological processes mapped by reactome** Biological processes associated with the shared genetic determinants between the multivariate genome-wide association statistics for mental health and the brain functional connectome as identified by the reactome toolbox. | | | | |
| --- | --- | --- | --- | --- |
| *Pathway name* | *No of genes involved* | *p-value* | *p-value (FDR)* | *Genes involved* |
| Axonal growth stimulation | 2 | 0.0005 | 0.2235 | NGFR; RHOA |
| Signaling by MST1 | 2 | 0.0014 | 0.2491 | MST1; MST1R |
| MECP2 regulates transcription factors | 2 | 0.0021 | 0.2491 | MEF2C |
| Axonal growth inhibition (RHOA activation) | 2 | 0.0026 | 0.2491 | NGFR; RHOA |
| p75NTR regulates axonogenesis | 2 | 0.0030 | 0.2491 | NGFR; RHOA |
| Respiratory electron transport, ATP synthesis by chemiosmotic coupling, and heat production by uncoupling proteins. | 5 | 0.0038 | 0.2595 | NDUFAF3; QRICH1; ATP5G1; LRPPRC |
| Inactivation, recovery and regulation of the phototransduction cascade | 3 | 0.0059 | 0.3361 | NMT1; CAMKMT |
| The phototransduction cascade | 3 | 0.0075 | 0.3361 | NMT1; CAMKMT |
| Non-integrin membrane-ECM interactions | 3 | 0.0082 | 0.3361 | LAMB2; DAG1 |
| Formation of ATP by chemiosmotic coupling | 2 | 0.0106 | 0.3361 | ATP5G1 |
| ECM proteoglycans | 3 | 0.0164 | 0.3361 | LAMB2; DAG1 |
| Laminin interactions | 2 | 0.0187 | 0.3361 | LAMB2 |
| Cristae formation | 2 | 0.0187 | 0.3361 | ATP5G1 |
| MET activates PTK2 signaling | 2 | 0.0198 | 0.3361 | LAMB2 |
| NFG and proNGF binds to p75NTR | 1 | 0.0199 | 0.3361 | NGFR |
| Defective POMGNT1 causes MDDGA3, MDDGB3 and MDDGC3 | 1 | 0.0199 | 0.3361 | DAG1 |
| EPHA-mediated growth cone collapse | 2 | 0.0210 | 0.3361 | EPHA3; RHOA |
| The citric acid (TCA) cycle and respiratory electron transport | 5 | 0.0224 | 0.3361 | NDUFAF3; QRICH1; ATP5G1; LRPPRC |
| Defective POMT2 causes MDDGA2, MDDGB2 and MDDGC2 | 1 | 0.0265 | 0.3361 | DAG1 |
| Defective POMT1 causes MDDGA1, MDDGB1 and MDDGC1 | 1 | 0.0265 | 0.3361 | DAG1 |
| Activation, myristolyation of BID and translocation to mitochondria | 1 | 0.0265 | 0.3361 | NMT1 |
| EGR2 and SOX10-mediated initiation of Schwann cell myelination | 2 | 0.0286 | 0.3361 | LAMB2; DAG1 |
| MAPK6/MAPK4 signaling | 3 | 0.0349 | 0.3361 | IGF2BP1; MAPKAPK5 |
| MET promotes cell motility | 2 | 0.0371 | 0.3361 | LAMB2 |
| NADE modulates death signalling | 1 | 0.0394 | 0.3361 | NGFR |
| p75NTR negatively regulates cell cycle via SC1 | 1 | 0.0394 | 0.3361 | NGFR |
| Cytosolic tRNA aminoacylation | 2 | 0.0448 | 0.3361 | QARS |
| Respiratory electron transport | 3 | 0.0454 | 0.3361 | NDUFAF3; QRICH1; LRPPRC |
| Regulation by TREX1 | 1 | 0.0458 | 0.3361 | TREX1 |
| Trafficking of myristoylated proteins to the cilium | 1 | 0.0458 | 0.3361 | ARL3 |
| Fanconi Anemia Pathway | 2 | 0.0497 | 0.3361 | FANCL; ATRIP |
